# Effects of the common polymorphism in the human aldehyde dehydrogenase 2 (*ALDH2*) gene on the lung

**DOI:** 10.1186/s12931-017-0554-5

**Published:** 2017-04-21

**Authors:** Aoi Kuroda, Ahmed E. Hegab, Gao Jingtao, Shuji Yamashita, Nobuyuki Hizawa, Tohru Sakamoto, Hideyasu Yamada, Satoshi Suzuki, Makoto Ishii, Ho Namkoong, Takanori Asakura, Mari Ozaki, Hiroyuki Yasuda, Junko Hamamoto, Shizuko Kagawa, Kenzo Soejima, Tomoko Betsuyaku

**Affiliations:** 1 Division of Pulmonary Medicine, Department of Medicine, Keio University School of Medicine, Shinjuku Ku, Shinanomachi 35, Tokyo, 160-8582 Japan; 2 Department of Pathology, Keio University School of Medicine, 35 Shinanomachi, Shinjuku-ku, Tokyo, 160-8582 Japan; 3 Beijing Chest Hospital, Beijing Tuberculosis and Thoracic Tumor Research Institute, Capital Medical University, Beijing, 101149 China; 4 Department of Pulmonary Medicine, Faculty of Medicine, University of Tsukuba, Ibaraki, 305-8575 Japan; 5 Department of Thoracic Surgery, Japanese Red Cross Ishinomaki Hospital, 71 Nishimichishita, Hebita, Ishinomaki, 986-8522 Japan

**Keywords:** ALDH2, Aldehyde, Epithelial cell, Basal cell, Club cell, Trachea, Lung, Stem cell, Mitochondria

## Abstract

**Background:**

Aldehyde dehydrogenases (ALDHs) play a major role in detoxification of aldehydes. High expression of ALDHs is a marker for stem cells of many organs including the lungs. A common polymorphism in *ALDH2* gene (ALDH2*2) results in inactivation of the enzyme and is associated with alcohol flushing syndrome and increased risk for cardiovascular and Alzheimer’s diseases and some cancers. The effect of this *ALDH2* polymorphism on the lung and its stem cells has not been thoroughly examined.

**Methods:**

We examined the association between the ALDH2*2 allele and lung function parameters in a population of healthy individuals. We also examined its association with the incidence of asthma and COPD in patient cohorts. We used the in vitro colony forming assay to detect the effect of the polymorphism on lung epithelial stem cells from both primary human surgical samples and Aldh2*2 transgenic (Tg) and *Aldh2* ^−/−^ mice. Response to acute and chronic lung injuries was compared between wild type (WT), Aldh2*2 Tg and *Aldh2* ^−/−^ mice.

**Results:**

In humans, the ALDH2*2 allele was associated with lower FEV1/FVC in the general population, but not with the development of asthma or COPD. Both the bronchial and lung epithelium carrying the ALDH2*2 allele showed a tendency for lower colony forming efficiency (CFE) compared to *ALDH2* allele. In mice, the tracheal epithelial thickness, nuclear density, and number of basal stem cells were significantly lower in *Aldh2* ^*−/−*^ and Aldh2*2 Tg adult mice than in WT. Electron microscopy showed significantly increased number of morphologically abnormal mitochondria in the trachea of *Aldh2* ^−/−^ mice. *Aldh2* ^−/−^ tracheal and lung cells showed higher ROS levels and fewer functional mitochondria than those from WT mice. No significant differences were detected when tracheal and lung epithelial stem cells were examined for their in vitro CFE. When exposed to chronic cigarette smoke, Aldh2*2 Tg mice were resistant to emphysema development, whereas influenza infection caused more epithelial damage in *Aldh2* ^−/−^ mice than in WT mice.

**Conclusions:**

*ALDH2* polymorphism has several subtle effects on the lungs, some of which are similar to changes observed during normal aging, suggesting a “premature lung aging” effect.

## Background

All organs and tissues in the body are continuously exposed to locally produced endogenous oxidants and aldehydes [1, 2]. Oxidants are generated during normal physiological processes and are reactive intermediates capable of damaging cellular components [1]. Aldehydes are the major end products of lipid peroxidation and can associate with proteins and nucleic acids to generate adducts, known to exert various harmful effects [2]. Luckily, most cells produce antioxidants and aldehyde dehydrogenases (ALDHs), enabling them to keep these harmful products under control and minimize their deleterious effects [3, 4]. Human lung, however, is additionally exposed to exogenous sources of oxidants and aldehydes from air pollution and cigarette smoke (CS). These factors seem to overwhelm lung defenses as aldehydes and oxidants in CS induce various harmful effects, including airway inflammation, cellular injury, DNA damage, and cytotoxicity [5–7]. High levels of *ALDHs* are observed in stem cells of many tissues and organs [8], including the proximal airways [9, 10]. The increased levels are considered to offer additional protection to stem cells against aldehydes. ALDH2 is one of the highest expressed ALDHs in the airways [9, 10]*.* ALDH2 concentration is prominently elevated in the bronchoalveolar lavage fluid of patients with chronic obstructive pulmonary disease (COPD) [11]. The main substrate of ALDH2 is acetaldehyde, an intermediary product during ethanol metabolism. It functions mainly in the mitochondria, which are also an important source of reactive oxygen species (ROS). Furthermore, ALDH2 seems to function as an antioxidant as its overexpression provides protection from oxidative stress, while its deficiency augments the stress [12, 13].

A single nucleotide polymorphism in *ALDH2* (termed ALDH2*2), rs671, results in the amino acid change Glu478Lys. This mutant allele has a dominant-negative effect, resulting in the complete or near-complete loss of ALDH2 enzymatic activity in individuals who are homozygous or heterozygous for the ALDH2*2 allele [14]. This polymorphism is very common in East Asians and affects almost half of the population [14]. Epidemiological and functional studies found that the ALDH2*2 allele is associated with facial flushing and increased pulse rate upon alcohol consumption [15], increased risk for cardiovascular diseases [16], late-onset Alzheimer’s disease [17], osteoporosis [18], and several alcohol-related cancers, including oropharyngolaryngeal, esophageal, stomach, and colon cancers [19].

However, despite distinctive lung exposure to both endogenous as well as exogenous aldehydes and oxidants, the effects of loss of ALDH2 function on the lungs of individuals with the ALDH2*2 polymorphism have not been studied extensively.

In this study, we extensively examined the effect of ALDH2 functional disturbance on lung histology and function in both humans and mice using in vitro and in vivo studies as well as a human genetic association study.

## Methods

### Human subjects for the genetic association study

To detect the overall effect of the *ALDH2* polymorphism on lung function in the general population, we conducted a cross-sectional association study on healthy volunteers (*n =* 967). Longitudinal data were also assessed for the annual decline of forced expiratory volume in 1 s (FEV1) over at least four years (*n =* 742). Additionally, to identify if the mutant allele is associated with the incidence of bronchial asthma or COPD, we genotyped a cohort of patients with bronchial asthma (*n =* 751) and COPD (*n =* 289) in comparison to the general population. Patients with bronchial asthma and COPD were recruited from the Tsukuba University Hospital and its affiliated hospitals [20–22]. Recruitment of the healthy volunteers was carried out as described previously [23]. They were recruited from the general population who visited the hospital for annual health checkups. Individuals with no evidence of pulmonary disease were included as healthy volunteers. Genomic DNA was extracted from the peripheral blood samples of all participants by an automated DNA extraction system (QuickGene-610 L; Fujifilm, Tokyo, Japan). Genotyping of patients with bronchial asthma and COPD was carried out by using the TaqMan Drug Metabolism Genotyping Assays. Genotyping of healthy volunteers was carried out by using the Illumina Human-Hap550v3 BeadChip assay (Illumina, San Diego, CA, USA) and the genotyping data of ALDH2*2 (rs671) was extracted using PLINK version 1.07 [23, 24]. Associations of the ALDH2*2 genotype with lung function data in healthy volunteers were analyzed by linear regression models and were adjusted for age, sex and smoking status in PLINK version 1.07. Chi-square tests were used to analyze the association between the ALDH2*2 genotype and the development of asthma and COPD.

### Collection of epithelial cells from human lung and bronchial samples

Human surgical samples were collected from patients with a lung pathology that was clinically indicated for surgical removal. Fifteen samples were from a “lobectomy”, three were from a “pneumonectomy”, and six were from a “lung segment” removal. An apparently healthy lung portion away from the tumor and a portion from the bronchial stump were excised, shipped overnight on ice and were processed immediately upon receipt.

Small portions were used for DNA and RNA extraction, and for paraffin embedding for histological assessment. The remaining tissues were used for epithelial cell retrieval. The lung tissue was finely minced and incubated at 37 °C in 10 U/mL elastase (Porcine Pancreatic, Elastin Products Company, Owensville, MO, USA) for 30 min. The suspension was passed through an 18G needle using a large syringe to help disperse the tissue pieces into single cells. The cell suspension was then filtered through a 100-μm strainer to remove the undigested clumps. RBCs were depleted by adding 4 mL of ACK lysis buffer. Up to 500 μL of DNase (Sigma Aldrich, St. Louis, MO, USA) was added to the cell pellet. Then cells were incubated in 0.25% trypsin/EDTA at 37 °C for 20 min to obtain a single cell suspension. Hematopoietic and endothelial cells were then depleted using anti-human CD45 and CD31 microbeads on the autoMACS cell separation system (Miltenyi Biotec, Cologne, Germany). The cells were stained with an anti-human EpCAM antibody (BD Biosciences, Franklin Lakes, NJ, USA) and the epithelial cells were sorted on a MoFlo sorter. Non-epithelial cells were plated on plastic flasks in DMEM for 2 h to isolate the fibroblasts. Unattached cells were discarded and the fibroblasts were allowed to proliferate to >90% confluence, and were then frozen for future use in co-cultures.

The portions from bronchi were cleaned under a dissecting microscope and were cut open, followed by incubation in 50 U/mL dispase for 2 h at room temperature. Detached epithelial sheets were scrubbed off the inner surface and were incubated in 0.25% trypsin/EDTA at 37 °C for 30 min to obtain a single cell suspension. These cells were then referred to as primary human bronchial epithelial cells (HBECs), and cultured as such or stained for a basal cell marker (ITGA6, NGFR, or CD44) followed by sorting for basal cells.

### In vitro 3D human organoid culture experiments

Bronchial epithelial cells were resuspended in MTEC/Plus medium and were mixed 2:1 with growth factor-reduced Matrigel (BD Biosciences). Lung cells were co-cultured with human lung fibroblasts in transwell inserts as previously described [9, 10]*.*

Some culture wells were treated with the ALDH2 activator, Alda-1 [25] (Merck Millipore, Darmstadt, Germany), at 100–300 μM and/or H_2_O_2_ at 300–400 μM as a source of ROS. The growing colonies were imaged and were visually counted on day 14 and/or 21. Colonies in the inserts were either embedded in Histogel (Thermo Scientific, Waltham, MA, USA) followed by paraffin for histological assessment, or were digested with dispase and trypsin to single cells. Single cells were either passaged or were used for RNA extraction.

Genotyping of human surgical samples was conducted using 2 sets of PCR primers with DNA isolated from patient lung samples as previously described [26]. Genotypes were further confirmed by the TaqMan® genotyping assay (TaqMan Drug Metabolism Genotyping Assays -ALDH2, Drug Metabolism Genotyping Assay mix, Life Technologies Corporation, Waltham, MA, USA).

### qRT-PCR

mRNA expression of other lung-expressed ALDHs and several antioxidant genes was assessed by qRT-PCR. The following primers were used on a Step-One ABI cycler using SYBR green.

*ALDH1A1*: 5′-TGGCTGATTTAATCGAAAGAGAT-3′,

5′-TCCACCATTCATTGACTCCA-3′,

*ALDH3A1*: 5′-GGGAAGCAGGGTCCTTAAAT-3′,

5′-CGCTGATCTTGCTCATGG-3′,

*HMOX-1*: 5′-GGCAGAGGGTGATAGAAGAGG-3′,

5′-AGCTCCTGCAACTCCTCAAA-3′,

*PRDX1*: 5′-AGGCCTTCCAGTTCACTGAC-3′,

5′-CAGGCTTGATGGTATCACTGC-3′,

*NQO1*: 5′-CAGCTCACCGAGAGCCTAGT-3′,

5′-GAGTGAG CCAGTACGATCAGTG-3′,

*NRF2*: 5′-GCGACGGAAAGAGTATGAC-3′,

5′-GTTGGCAGATCCACTGGTTT-3′,

*GAPDH*: 5′- GAGTCAACGGATTTGGTCGT-3′,

5′-TTGATTTTGGAGGGATCTCG-3′.

Gene expression was expressed as ratios to *GAPDH*.

### Western blotting

Small pieces of human lung samples were homogenized and lysed with cell lysis buffer and followed by measurement of protein concentrations. Equal amounts of protein were fractionated by electrophoresis and then transferred to PVDF membranes. The membranes were then incubated with antibodies against NRF2 (Santa Cruz), ALDH1A1 (Abcam), and ALDH3A1 (Santa Cruz) followed by incubation with secondary antibodies. For the detection of specific protein bands, the membranes were incubated in LumiGLO reagent and peroxide (Cell Signaling Technologies, Danvers, MA, USA), and then exposed to X-ray films.

### Animals

We used two different types of mice with disturbed ALDH2 function; Aldh2*2 transgenic (Tg) mice were generated by the pronuclear injection of a plasmid carrying mouse Aldh2*2 cDNA with a single nucleotide mutation at the same position as that of the human ALDH2*2 polymorphism, under the control of CAG promoter [27] and *Aldh2* ^*−/−*^ mice, in which the endogenous *Aldh2* gene is replaced by the neomycin-resistance gene [28]. The wild type (WT) littermates were used as controls. Trachea and lung paraffin sections from WT, Aldh2*2 Tg, and *Aldh2* ^*−/−*^ mice were collected from newborn, adult, and aged mice and stained with hematoxylin and eosin (H&E). Tracheas were examined for epithelial thickness at 3 different locations. Because of natural variation in epithelial thickness between tracheal non-cartilaginous posterior portion and the rest of the circumference, and between uppermost and lowermost parts, we restricted our measurements and comparisons to the supracartilaginous regions of the sides of tracheal portion between cartilaginous rings 4 and 8. Four to six mice were analyzed per group. Nuclear densities were calculated by counting number of nuclei per a 100 μm. Quantification for percentage of cellular types in all groups was performed by counting from immunostained sections.

### Immunostaining

Human surgical samples and mice samples were fixed with 4% paraformaldehyde and embedded in paraffin. Tissue sections (6-μm thickness) were prepared and immunostained as described previously [29]. The primary antibodies used included rabbit K5 (Covance, Princeton, NJ, USA), goat CC10 (SCGB1A1) and Pro-surfactant protein C (SPC) (Santa Cruz Biotechnology), TTF1 (Abcam), mouse MUC5AC (Thermo Scientific), and mouse acetylated *β*-tubulin (Sigma) for the identification of basal, club (Clara), alveolar type II, goblet, and ciliated cells. Interleukin (IL)-1β (Abcam), Gr-1 (BD), Peroxiredoxin 1 (Prdx1) (Abcam) and Hemagglutinin (Abcam) were used for characterization of the injury models. The appropriate Alexa-Fluor coupled secondary antibodies were used in double and triple stained sections. The nuclei were stained with DAPI and the slides were then examined by fluorescence microscopy using a Zeiss AxioImager microscope (Carl Zeiss).

### Transmission electron microscopy (TEM)

Tracheas were cut between the 5^th^ and 8^th^ tracheal cartilaginous rings. All samples were fixed in 2.5% glutaraldehyde/0.1 M phosphate buffer (pH 7.4) for 2–4 h. After post-fixation in 1.5% osmium tetroxide/ 0.1 M phosphate buffer (pH 7.4) for 1 h, the samples were dehydrated in ethanol, treated with propylene oxide, then embedded in an epon resin, which was polymerized at 60 °C for 2–3 days. Semi-thin sections were stained with toluidine blue to select an optimal area of the epithelium with straight basement membrane and without artifacts. Ultra-thin sections were mounted on a nickel mesh and were electron-stained with uranyl acetate and lead citrate. The sections were then examined under a transmission EM, JEM 1400 (JEOE, Tokyo, Japan).

### Mitochondrial functional assessments

#### 1-Extracellular flux bioenergetics assay

Mouse tracheal epithelial cells (MTEC) were collected from at least 4 *Aldh2* ^*−/−*^ and WT mice. As this assay requires cells to be attached, extracellular flux 24-well culture plates (Seahorse Bioscience) were coated with 50 μg/mL collagen (BD Biosciences). These were then seeded in triplicates with 120,000 cell/well and were incubated for 2 days at 37 °C. The Oxygen Consumption Rate (OCR) and Extracellular Acidification Rate (ECAR) were measured in all wells according to the manufacturer’s instructions (Seahorse Bioscience) and as described previously [9, 30].

#### 2-Examination of mitochondrial inner membrane protein

The mitochondrial membrane protein Tim23 (Santa Cruz Biotechnology) was examined by immunostaining of histological sections from mouse lung and tracheal tissues and in vitro colonies.

#### 3-Measurment of ATP production

Using the CellTiter-Glo® Luminescent Cell Viability Assay (Promega, Fitchburg, WI, USA) in an opaque-walled 96 well plate, triplicate wells of serum-containing culture medium without cells, and a gradient of cell numbers from the lung and tracheal epithelium from the different mice were tested: 1000, 5000, 25,000, 50,000, and 100,000 cells in 100 μL vol according to the manufacturer’s protocol. Luminescence was recorded on a plate reader at 490 nm.

#### 4-Quantification of functional mitochondria

MitoTracker® Red FM and/or MitoTracker® Red CMXRos (Promega) were used according to the manufacturer’s protocol. MTECs and lung cells were collected from WT, Aldh2*2 Tg, and *Aldh2* ^*−/−*^ mice. From each sample, 150,000 cells were stained in suspension, either examined by flow cytometry or cytospun, and examined by fluorescence microscopy.

#### 5-Quantification of mitochondrial ROS levels

MitoSOX Red (Promega) was used according to the manufacturer’s protocol and 150,000 cells were stained and examined by flow cytometry or fluorescence microscopy.

The cells that were used for fluorescence microscopy analysis were cytospun onto glass slides. The cells were fixed by dispensing 100 μL of acetone onto the slide and allowed to air-dry. DAPI was then added to the cells. The cells were covered with a cover slip.

### In vitro 3D mouse organoid culture experiments

Collection of MTEC and whole lung epithelium, isolation of lung fibroblasts and their co-culture in Matrigel were essentially performed as described previously [29]. The medium used, treatment with H_2_O_2_ and/or Alda-1, quantification and processing of colonies at the end point were performed in a manner similar to the human organoid culture described above.

### In vivo injury models

#### 1-Chronic injury with CS

Ten adult Aldh2*2 Tg mice and their WT littermates were exposed to mainstream CS using commercially available cigarettes (Marlboro, 12 mg tar/1.0 mg nicotine) as previously described [31]. Exposure to 4% CS was given for one hour/day and 5 days/week for 16 weeks. Age-matched control mice for each group were exposed to air over the same period. Emphysema was assessed in the living animals by micro CT and after euthanasia on the histological sections, and was quantified using mean linear intercepts (Lm) [31].

#### 2-Acute injury with H1N1 influenza and polidocanol

Eight WT mice and seven *Aldh2* ^*−/−*^ mice were infected through the nose with a sub-lethal dose of influenza H1N1 PR8 (100 pfu/50 μL). Survival and body weight were recorded daily. The lungs were collected on day 8, embedded in paraffin, and the sections were stained with H&E and immunostained for epithelial and hematopoietic (inflammatory) cell markers like SPC, CC10, β-tubulin, and CD45. Viral load was assessed by staining for hemagglutinin.

Polidocanol has been previously used to induce acute injury of the airway epithelium. Ten microliters of 2% polidocanol were administered to mice anesthetized with ketamine/xylazine [32]. However, in our hands, this dosing strategy resulted in more than 50% mortality. Thus, we performed preliminary experiments to optimize the administration technique. We optimized the route of administration (intra-nasal, intra-tracheal via oral intubation or through a neck incision), the device used for polidocanol delivery (insulin syringe or micro-sprayer), the volume of polidocanol (10, 15, and 20 μL), the concentration of polidocanol (0.5, 1, and 2%), the mode of anesthesia (ketamine i.p., isoflurane inhalation, combination of ketamine i.p. and isoflurane inhalation, isoflurane inhalation with continuous O_2_), and the time points for sample collection (12 h, 24 h, 48 h, 5 days, and 7 days).

### Statistics

Data from all samples from each group are expressed as mean ± SD. The quantification of CFE and cell types was performed by visual counting under a microscope and digital images from at least 3 triplicates and 3 independent experiments. *T* test was used for pairwise comparisons and a *P* value less than 0.05 was considered significant.

## Results

### Japanese individuals with the ALDH2*2 genotype have lower lung function parameters

Healthy volunteers (*n =* 967) were genotyped for the *ALDH2* polymorphism then were compared for their lung function parameters based on their genotype. The longitudinal data for the annual decline of forced expiratory volume in 1 s (FEV1) over at least four years were available from 742 individuals. The incidence of the *ALDH2* polymorphism was also studied in bronchial asthma (*n =* 751) and COPD (*n =* 289) patient cohorts in comparison to the general population. The frequency of the ALDH2*2 allele in these three populations ranged between 41.1–44.8%, consistent with previous reports for the Japanese population and in keeping with the Hardy–Weinberg equilibrium. The clinical characteristics of all participants are shown in Table 1.

**Table 1:** Characteristics of healthy human participants, asthmatic and COPD patients

	Healthy volunteers (*n =* 967)	Bronchial asthma (*n =* 751)	COPD (*n =* 289)
Age (years)	50.0 ± 9.1	57.0 ± 16.8	74.7 ± 8.6
Female (%)	526 (54.4%)	445 (59.3%)	31 (10.6%)
Smoking Index Group^a^, ^b^ 0	607 (62.8%)	481 (64.0%)	10 (3.5%)
1–200	126 (13.0%)	103 (13.7%)	9 (3.1%)
>200	234 (24.2%)	145 (19.3%)	269 (93.1%)
FVC (L)	3.22 ± 0.76	2.92 ± 0.94	2.65 ± 0.87
FEV1 (L)	2.68 ± 0.64	2.11 ± 1.06	1.25 ± 0.58
Predicted FEV1 (%)	93.3 ± 12.1	85.3 ± 22.7	53.4 ± 22.1
FEV1/FVC (%)	83.2 ± 5.2	71.0 ± 12.4	47.2 ± 12.4
FEV1 decline (mL/year)	23.4 ± 49.3 (*n =* 742)	NA	NA

^a^The Smoking Index Group is calculated by multiplying the average number of cigarettes smoked per day with the number of years of smoking

^b^Smoking history was not available for 22 and 1 asthma and COPD patients, respectively

The presence of an ALDH2*2 allele A is associated with a significantly lower FEV1/FVC than that of the WT *ALDH2* allele G within the group of healthy individuals (GG *vs.* GA *vs.* AA, β = −0.621, *p* = 0.0164). However, the presence of the ALDH2*2 allele A was not associated with predicted FEV1% (GG *vs.* GA *vs.* AA, β = −0. 424, *p* = 0.486) or the rate of annual FEV1 decline over the examined period of 4 years (GG *vs.* GA *vs.* AA, β = −1.122, *p* = 0.496). Additionally, the ALDH2*2 allele was not associated with the development of bronchial asthma or COPD (*p* = 0.42 and 0.747, respectively).

### Histological and functional assessment of human bronchial and lung epithelium revealed no major difference between individuals with *ALDH2* and ALDH2*2 genotypes

**Fig. 1:**
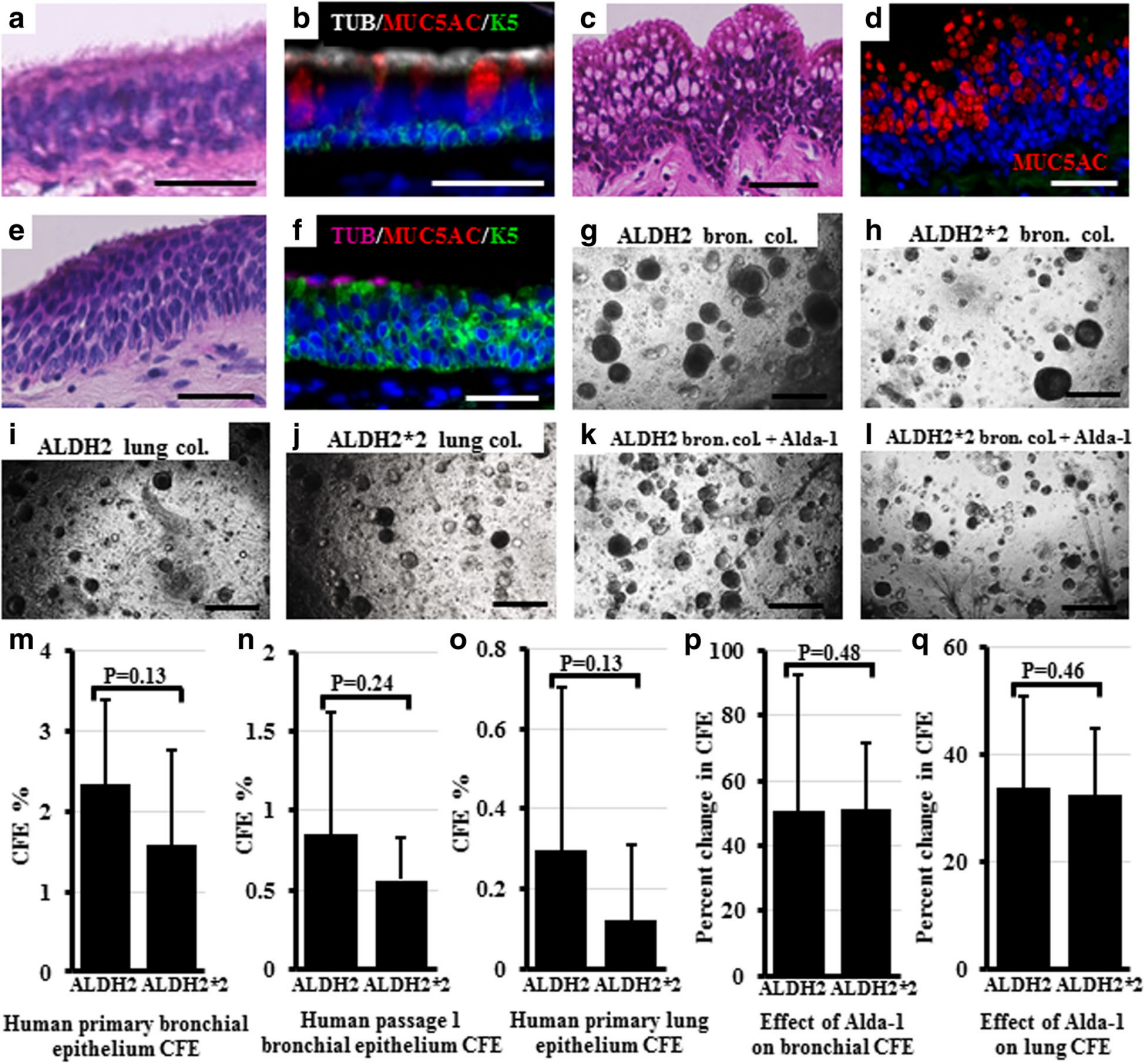
Histology and in vitro CFE of human samples with the *ALDH2/ALDH2* or ALDH2/ALDH2*2 heterozygous alleles. Bronchial sections from all individuals were stained with H&E and were immunostained for basal, goblet, and ciliated cell markers using antibodies against K5, MUC5AC, and *β*-tubulin, respectively. **a**-**f** show representative images for: normal epithelial lining (**a**, **b**) goblet cell hyperplasia **c**, **d** and basal cell hyperplasia (**e**, **f**). Bronchial and lung epithelial cells isolated from *ALDH2/ALDH2* and ALDH2/ALDH2*2 heterozygous samples were cultured in the in vitro 3D- stem cell colony forming assay. Representative images are shown for the bronchial colonies (**g**, **h**) and lung colonies (**i**, **j**) from the *ALDH2/ALDH2* and ALDH2/ALDH2*2 heterozygous samples, respectively. **k**, **l** show representative images for the inducing effect of ALDH2 activation by Alda-1 treatment on the bronchial CFE (compare to **g** and **h**). CFE of the primary and clonally passaged cells were calculated for all the samples examined (**m**-**o**). The effect of ALDH2 activation on CFE was calculated for each sample from parallel-treated culture wells. The percentage of increase in CFE in comparison to the untreated wells is shown (**p**, **q**). ALDH2: *ALDH2/ALDH2*, ALDH2*2: ALDH2/ALDH2*2, Bron: bronchial, Col: colonies. Scale bars: (**a**-**f**) 20 μm; (**g**-**l**) 500 μm. **b**, **d**, and **f**: Nuclei were stained with DAPI (*blue*)

The human surgical samples assessed, included 26 lung and 23 bronchial samples. DNA was isolated from all samples and genotyped for the *ALDH2* polymorphism (with *ALDH2/ALDH2* alleles i.e. has functional ALDH2, heterozygous ALDH2/ALDH2*2, or homozygous for the ALDH2*2 allele i.e. with non-functional ALDH2). All human samples examined were either *ALDH2/ALDH2* or ALDH2/ALDH2*2 heterozygous and no ALDH2*2 homozygous samples. Fixed and embedded bronchial portions were histologically assessed for epithelial abnormalities. Of 13 bronchial samples with the *ALDH2* alleles, three samples showed goblet cell hyperplasia. Of 10 ALDH2/ALDH2*2 heterozygous bronchial samples, two showed basal cell hyperplasia, one showed goblet cell hyperplasia, and one showed both goblet and basal cell hyperplasia (Fig. 1a-f). Smoking history was not associated with hyperplasia development in both genotypes. Then, to detect a potential functional impairment on stem cells, both bronchial and lung epithelial stem cells were examined using the in vitro 3D colony forming assay [9, 10]*.* The CFE of primary and passaged epithelial cells of *ALDH2/ALDH2* and ALDH2/ALDH2*2 heterozygous bronchial and lung samples was compared (Fig. 1g-l). The average primary bronchial CFE from 8 *ALDH2/ALDH2* samples was 2.33%, while that from 5 ALDH2/ALDH2*2 heterozygous samples was only 1.58% (Fig. 1m). When the colonies were passaged to examine clonality, CFE at passage 1 in 6 *ALDH2/ALDH2* samples was 0.86%, and that in 4 ALDH2/ALDH2*2 heterozygous samples was 0.56% (Fig. 1n). The primary lung CFE in 11 *ALDH2/ALDH2* samples was 0.3%, and that in 6 ALDH2/ALDH2*2 heterozygous samples was 0.12% (Fig. 1o). Although the average CFE of both bronchial and lung samples in samples with the *ALDH2* genotype tended to be higher than that with the ALDH2*2 genotype, the difference was not statistically significant. ALDH2 activation using a small molecule activator, Alda-1 [25], successfully increased the CFE of both bronchial and lung ALDH2/ALDH2*2 heterozygous epithelium by approximately 50% and 34% of their original efficiency, respectively. However, Alda-1 treatment of *ALDH2/ALDH2* bronchial and lung epithelium produced a similar respective induction of 51% and 32.5% (Fig. 1k, l, p, q). Analysis of the differentiation profile of bronchial and lung colonies from both genotypes revealed no difference.

### Loss of ALDH2 function is associated with downregulation of antioxidant genes in the human lungs, but no effect was detected on other ALDHs

**Fig. 2:**
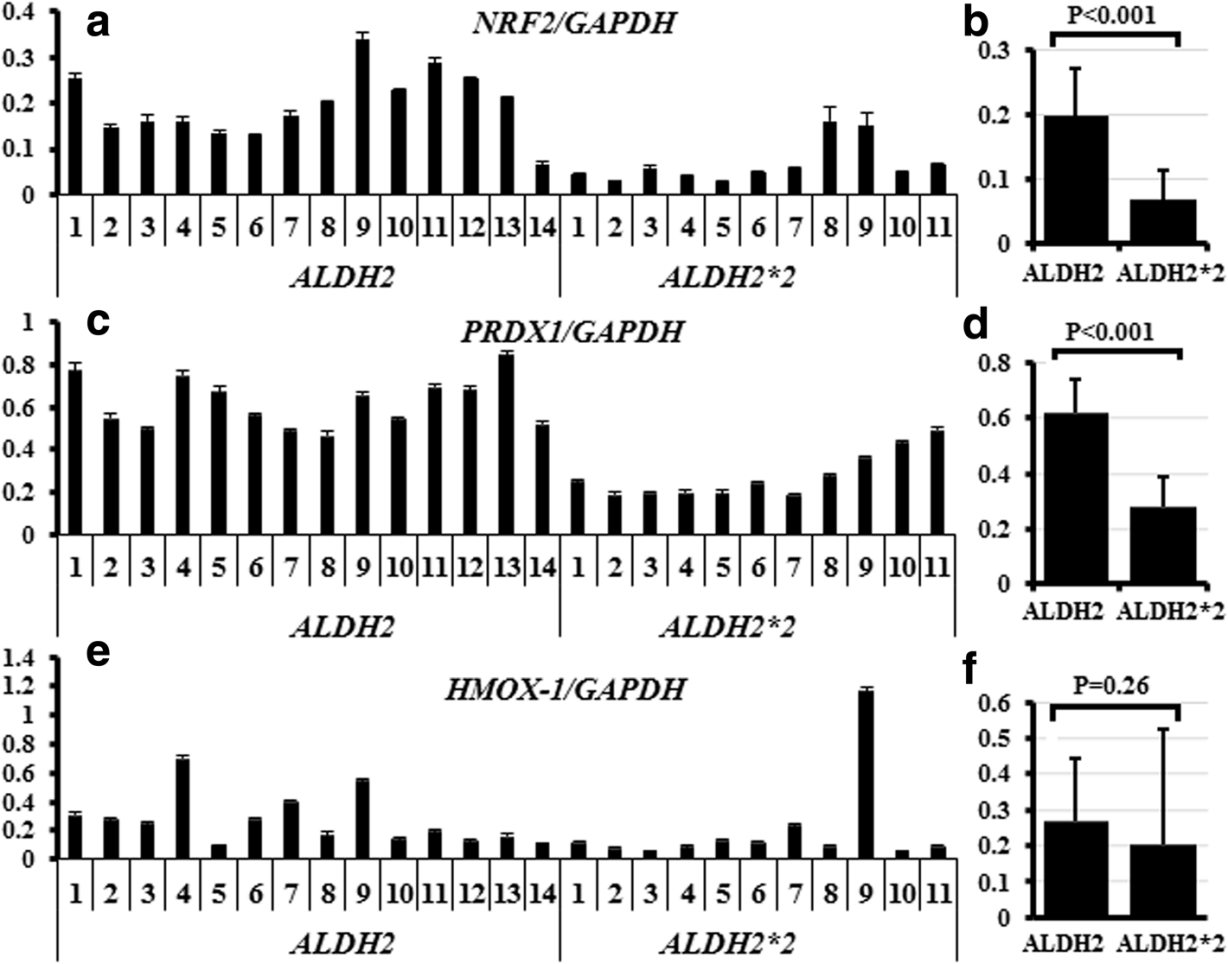
Effect of ALDH2 functional disturbance on the expression of antioxidant genes. RNA extracted from all human samples was examined to determine the expression level of several antioxidant genes. Expression of *NRF2* (**a**, **b**) and *PRDX1* (**c**, **d**) was significantly higher in *ALDH2/ALDH2* than in the ALDH2/ALDH2*2 heterozygous samples. *HMOX-1* expression showed a similar trend but the differences were non-significant (**e**, **f**)

To examine if the absence of ALDH2 activity in the lungs results in compensatory upregulation or downregulation, or shows no effect on other functionally relevant genes, we examined the expression of other lung-expressed ALDHs and antioxidant genes in all human samples and analyzed their levels in individuals with *ALDH2/ALDH2* versus ALDH2/ALDH2*2 heterozygous genotypes using RT-PCR. The expression of antioxidant genes, *NRF2* and *PRDX1*, were significantly lower in the ALDH2/ALDH2*2 heterozygous than in the *ALDH2/ALDH2* (Fig. 2a-d). *HMOX-1* showed a trend towards lower expression but the difference was not significant (Fig. 2e, f). *ALDH1A1* and *ALDH3A1*, and the antioxidants, *NQO1* and *TXNRD1*, showed no significant difference. ALDH1A1 and ALDH3A1 expression was confirmed at the protein level using western blotting. The expression of these proteins varied among samples and no significant difference was observed between the *ALDH2/ALDH2* and ALDH2/ALDH2*2 heterozygous samples (data not shown).

### Tracheas of adult mice with disturbed Aldh2 function display fewer basal cells and lower epithelial cellular density than those of WT mice

**Fig. 3:**
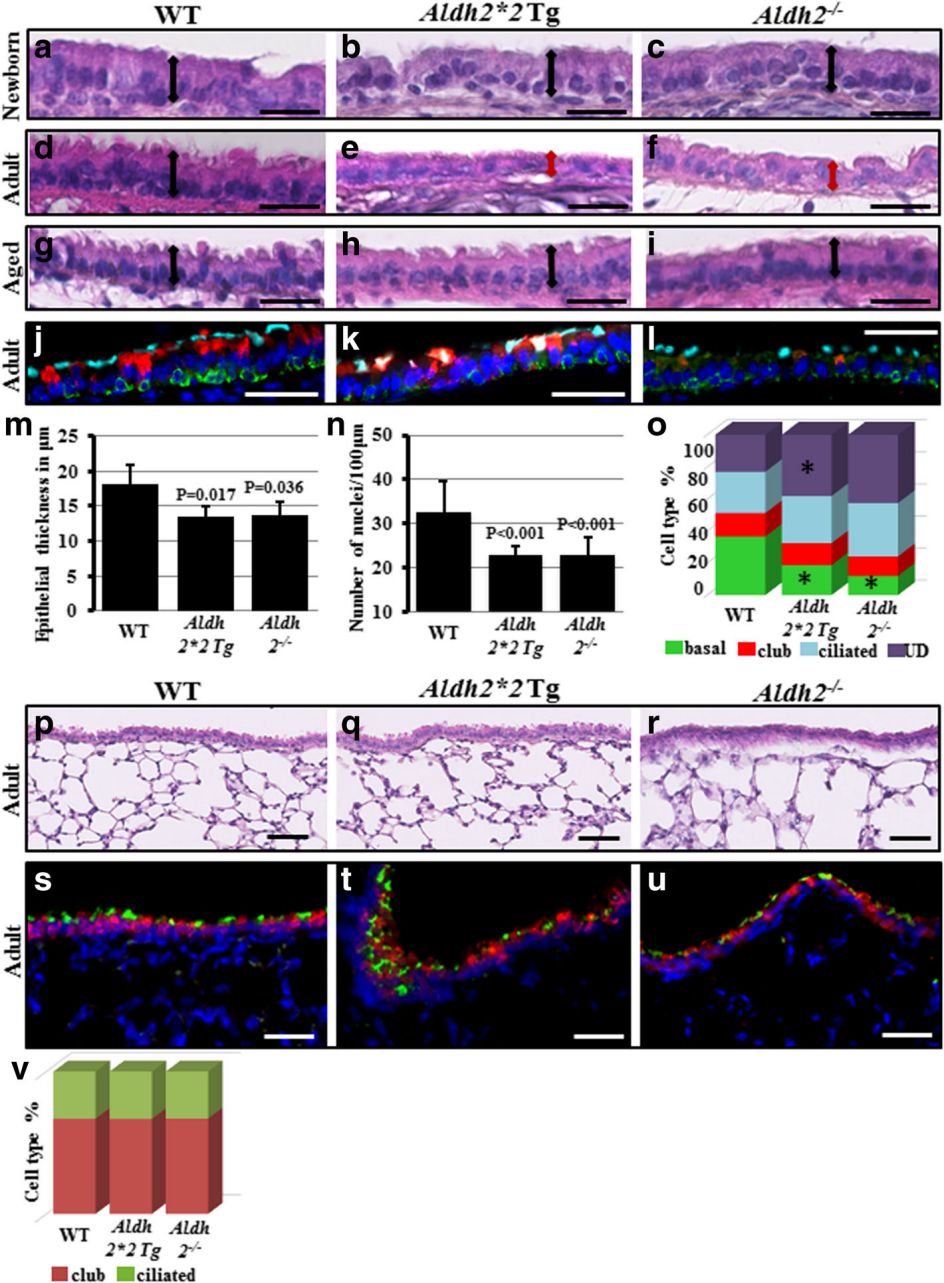
Tracheal and lung histology of WT, Aldh2*2 Tg, and *Aldh2* ^*−/−*^ mice. (**a**-**i**) Trachea paraffin sections from WT, Aldh2*2 Tg, and *Aldh2* ^*−/−*^ mice collected from newborn, adult, and aged mice were stained with H&E and examined for epithelial thickness and nuclear density, *n =* 4-6 mice/group. Two-headed arrows show epithelial thickness (**a**-**i**). Similar tracheal sections were immunostained for basal (K5-green), club (Clara) (CC10-red), and ciliated cell (*β*-tubulin-cyan) markers to quantify the tracheal cellular subtypes. Nuclei were stained with DAPI (blue) (**j**-**l**). Quantification of tracheal surface epithelial thickness (**m**) and nuclear density (**n**) from all three groups. **o** Quantification for percentage of tracheal cellular types in all groups. UD: Undetermined/Intermediate cells. *indicates significant P value compared to WT. Lung paraffin sections from adult WT, Aldh2*2 Tg, and *Aldh2* ^*−/−*^ mice were stained with H&E and examined for airway and parenchymal abnormalities (**p**-**r**). Similar lung sections were immunostained for club (Clara) (CC10-red) and ciliated cell (*β*-tubulin-green) markers to quantify the intrapumonary airway cellular subtypes. Nuclei were stained with DAPI (blue) (**s**-**u**). **v** Quantification for intrapumonary airway percentages of club/ciliated cells in all mice groups. Scale bars: **a**-**i**: 25 μm, **j**-**l**: 50 μm

To further characterize the effect of the *ALDH2* loss-of-function polymorphism on lung epithelial development, stem cell function, and repair after injury, we used two different types of mice with disturbed Aldh2 function. An *Aldh2* ^*−/−*^ mouse, which expresses no Aldh2 protein [28] and an Aldh2*2 Tg mouse, which possesses an intact endogenous *Aldh2,* but overexpresses the Aldh2*2 (mutant) gene and protein. This non-functional protein interferes with the functions of the endogenous Aldh2 in a “dominant negative effect”, mimicking the effect of the human *ALDH2* polymorphism [27]. We histologically compared the naïve mouse tracheas among WT, Aldh2*2 Tg, and *Aldh2* ^*−/−*^ mice at birth, adult, and old age (Fig. 3a-i). Interestingly, significant differences in the tracheal epithelia were observed only in adult mice, but not in the newborn or old aged mice. The tracheal epithelia were thinner and showed lower cellular density in Aldh2*2 Tg and *Aldh2* ^*−/−*^ mice than in WT mice (Fig. 3d-f, quantified in m-n).

When the cellular subtypes of adult tracheas from all groups were analyzed to explain the decrease in epithelial thickness and cellular density in Aldh2*2 Tg and *Aldh2* ^*−/−*^ mice, only the basal, but not the club or ciliated cells, showed a significantly lower ratio in Aldh2*2 Tg and *Aldh2* ^*−/−*^ mice than in WT mice (Fig. 3j-l, quantified in o). The decrease in the basal cell ratio was compensated for by an increase in ciliated and unidentified (intermediate/undetermined) cells, but not by the club cells. Basal cells are the stem cells in mouse trachea, capable of both self-renewal and differentiation into club and ciliated cells; the role of the unidentified (intermediate/undetermined) cells is not well understood [33]. These findings suggest that Aldh2 is involved in regulating the self-renewal and differentiation of basal stem cells in vivo. On the other hand, histological examination of the lung parenchyma and intra-pulmonary airways from all the adult mouse groups showed no significant difference compared to those of WT mice (Fig. 3p-r). Intrapulmonary airways, which contain no basal cells and are lined mainly with club cells and ciliated cells, were immunostained and cellular subtypes were examined at the level of proximal bronchi, mid-level bronchi, and terminal bronchioles in all mice genotypes and no significant differences were detected among them (Fig. 3s-u, quantified in v). The alveolar compartments showed no abnormality as well.

### Loss of Aldh2 results in marked increase in morphologically abnormal mitochondria in basal and club cells of mouse trachea

**Fig. 4:**
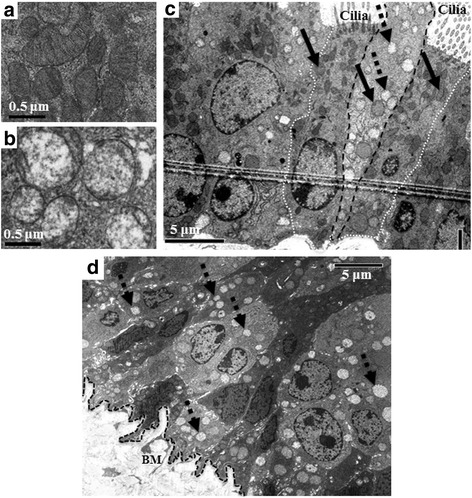
Detection of cells with a high number of morphologically abnormal mitochondria. Sections from WT, Aldh2*2 Tg, and *Aldh2* ^*−/−*^ adult mice as well as from aged WT mice were examined with TEM for the presence of cellular ultrastructure abnormality. Compared to the typical shaped normal mitochondria (**a**), morphologically abnormal mitochondria, with rounded shape, complete lack of cristae, and a marked decrease in matrix density were detected in all groups (**b**). Some cells showed most mitochondria of the abnormal type (dotted arrows) and a few mitochondria of the normal type (solid arrows) in club (dashed black line) and basal cells of all mice, but not in their ciliated cells (dotted white line) (**c**) (**a**, **b**, and **c** are representative photos from WT). *Aldh2* ^*−/−*^ tracheas showed a large number of cells with >80% mitochondria being abnormal (*dotted arrows*) (**d**). BM: Basement membrane

The relation between mitochondrial morphology and its respiratory activity has been identified for a long time [34]. Morphological differences between the mitochondria of club cells and ciliated cells have been described and are attributed to the high energy requirement of ciliated cells, resulting in their mitochondria having more cristae and a scanty matrix [35, 36]. In contrast, pathological conditions like hypoxia or increased oxidative stress result in “reduced-density” of the mitochondrial matrix [37, 38]. As ALDH2 is a mitochondrial enzyme whose deficiency augments oxidative stress [12, 13], we examined the effect of its deficiency on the mitochondrial morphology in airway epithelial cells, using TEM. In the tracheas of WT adult mice, the previously described morphological differences between the mitochondria in club and ciliated cells were observed (i.e., fewer cristae and expanded matrix in club cells) [35, 36]. In this study, we detected the presence of a previously unknown minor population of “abnormal” mitochondria, which showed near complete lack of cristae and a marked decrease in matrix density in all groups (Fig. 4a-b). We found these abnormal mitochondria in the club cells and basal cells, but not in ciliated cells (Fig. 4c). In most club and basal cells of the WT trachea, these abnormal mitochondria represented less than 20% of total mitochondria per cell. However, some cells had most of their mitochondria showing this abnormal morphology. We quantified these abnormal mitochondria in a large number of cells from all groups to identify whether or not they were associated with the *Aldh2* genotype. We set a threshold of 80% abnormal mitochondria per cell to count a cell as a “cell with abnormal mitochondria”. Interestingly, we observed a moderate increase in the percentage of these abnormal mitochondria per cell in the club and basal cells of the Aldh2*2 Tg and aged WT mice, but this increase was not as high as our set threshold of 80%. Surprisingly, many of the club and basal cells in *Aldh2* ^*−/−*^ mice showed these abnormal mitochondria beyond the 80% threshold and the number of these cells was strikingly high (Fig. 4d) (Table 2).

**Table 2:** Quantification of tracheal epithelial cells with abnormal mitochondria

	Basal cells	Club cells	Ciliated cells
WT	17% (*n =* 30)	14% (*n =* 66)	0% (*n =* 63)
WT (aged)	24% (*n =* 34)	11% (*n =* 61)	0% (*n =* 50)
Aldh2*2 Tg	19% (*n =* 31)	13% (*n =* 61)	0% (*n =* 53)
*Aldh2* ^*−/−*^	88% (*n =* 41)	96% (*n =* 75)	14% (*n =* 65)

Percentage of cells with more than 80% of its mitochondria abnormal within a cell type

(*n =* Number of cells examined per group)

### Functional assessment of mitochondria in the tracheal epithelium and in whole lung cells

**Fig. 5:**
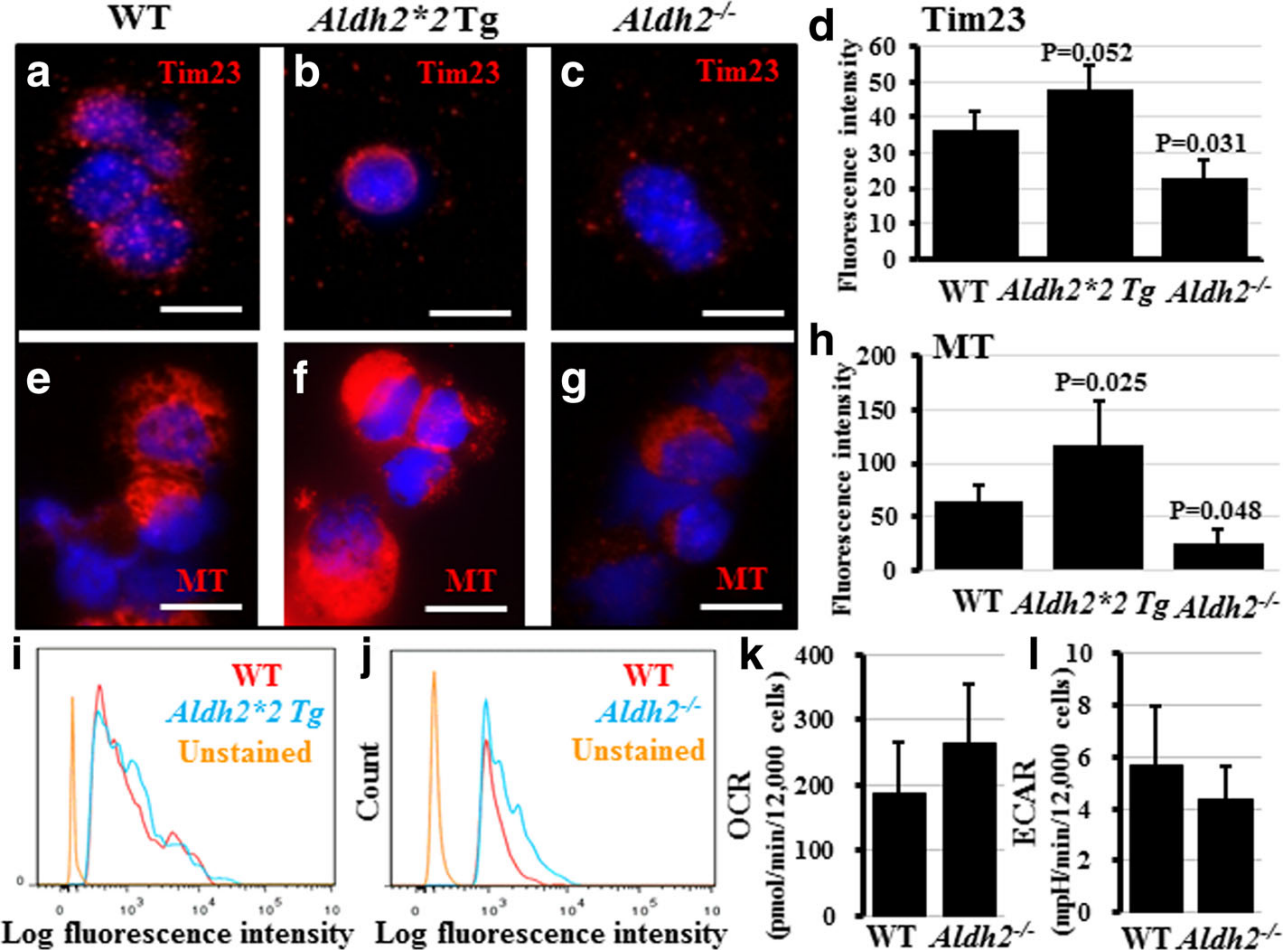
Functional and quantitative assessment of mitochondria in mouse tracheal and lung epithelium. MTECs and lung cells were collected from WT, Aldh2*2 Tg, and *Aldh2* ^*−/−*^ mice, *n =* 4-5 mice/genotype. Cells were immunostained for the mitochondrial membrane protein, Tim23. No difference in fluorescence intensity was observed between WT (**a**) and Aldh2*2 Tg (**b**), but MTECs from *Aldh2* ^*−/−*^ mice showed significantly lower fluorescence intensity (**c**). Quantification of fluorescence intensity from multiple cells from each genotype is shown in (**d**). Cells were also stained for MitoTracker (MT) probes and examined by fluorescence microscopy and flow cytometry. In comparison to WT cells (**e**), lung cells from Aldh2*2 Tg mice (**f**) had a higher fluorescence intensity, while the *Aldh2* ^*−/−*^ mice (**g**) showed lower fluorescence intensity. Quantification of fluorescence intensity from multiple cells from each genotype is shown in (**h**). Not Aldh2*2 Tg (**i**) but *Aldh2* ^*−/−*^ (**j**) showed a higher level of mitochondrial ROS than WT mice did. MTECs from WT and *Aldh2* ^*−/−*^ mice were examined using a flux analyzer to measure and compare OCR (**k**) and ECAR (**l**) as indicators of the cellular mitochondrial respiration and glycolysis. No significant difference was observed between WT and *Aldh2* ^*−/−*^ cells. **a**-**c**, **e**-**g** scale bars: 10 μm, nuclei were stained with DAPI (*blue*)

The finding of a considerable number of morphologically abnormal mitochondria in the tracheal cells of *Aldh2* ^*−/−*^ and, to a lesser extent, in Aldh2*2 Tg and WT old mice prompted us to conduct several mitochondrial functional assessments in these mice. We first assessed the amount of mitochondria in the tracheal and lung cells in the different mouse strains by immunostaining for the translocase of inner mitochondrial membrane, Tim23. We then examined the quantity of “functional” mitochondria using two different MitoTracker probes, which stain only the functional mitochondria with active membrane potential. Surprisingly, the tracheal and lung cells in *Aldh2* ^*−/−*^ mice showed fewer mitochondria (total and functional) than those in the WT, whereas the Aldh2*2 Tg cells contained more mitochondria than the WT (functional) (Fig. 5a-h). Secondly, we measured mitochondrial ROS production using MitoSOX. Compared to WT control, ROS level was higher in tracheal epithelial cells from the *Aldh2* ^*−/−*^ mice, but not from the Aldh2*2 Tg mice (Fig. 5i-j). Finally, we evaluated the energy production efficiency by exploiting the CellTiter-Glo Assay for measurement of ATP levels, and by measuring the overall contribution of the two major energy production pathways, the oxidative phosphorylation (mitochondrial respiration) pathway and glycolysis, in cellular respiration using the extracellular flux bioenergetics analyzer. No significant difference was observed in the ATP levels of the tracheal or lung cells among the groups (ATP levels in the Aldh2*2 Tg and *Aldh2* ^−/−^ lungs were 90% and 103% and in their MTEC were 97% and 94% of that in the cells of WT mice, respectively). Comparing the OCR and ECAR in WT and *Aldh2* ^*−/−*^ adult mice, MTECs showed no significant difference (Fig. 5k-l).

### Assessment of tracheal basal stem cell function in the in vitro 3D colony forming assay reveals no significant difference between the WT, Aldh2*2 Tg, and *Aldh2*^*−/−*^ mice

**Fig. 6:**
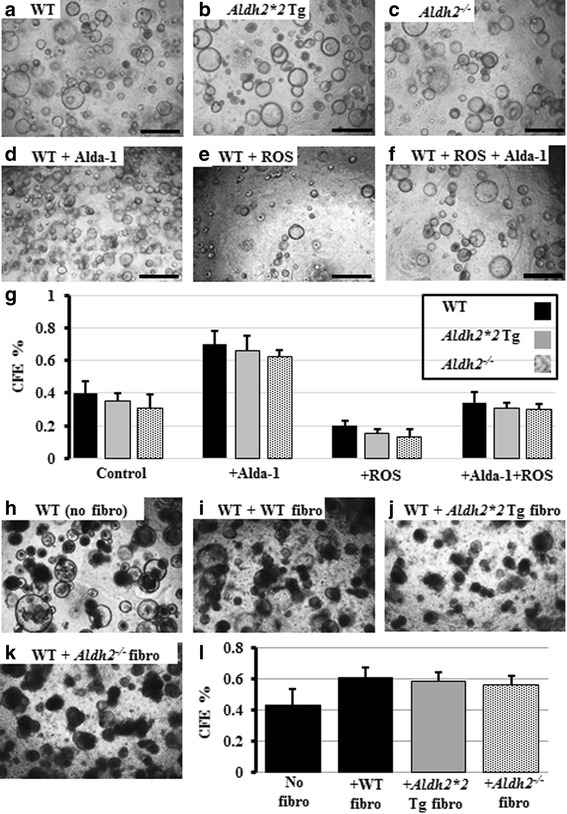
In vitro MTEC CFE of WT, Aldh2*2 Tg, and *Aldh2* ^*−/−*^ mice. MTECs from all groups were collected and cultured in the 3D stem cell colony forming assay (**a**-**c**) and parallel culture wells were treated with H_2_O_2_, and/or the Aldh2 agonist, Alda-1. Representative images of MTECs from WT mice after treatments are shown in (**d**-**f**). Quantification of all wells from all groups is shown in (**g**). WT MTECs were cultured alone (**h**) or co-cultured with lung fibroblasts isolated from WT, Aldh2*2 Tg, and *Aldh2* ^*−/−*^ mice (**i**-**k**). **l** Quantification of CFE from all co-culture wells

Next, we wanted to test whether the histological changes observed in the tracheal basal cells and the abnormalities in mitochondrial morphology and function that were detected in the tracheal basal and club cells of the Aldh2*2 Tg and *Aldh2* ^*−/−*^ mice negatively influenced the ability of basal cells to act as stem cells in vitro. We therefore isolated MTECs from all groups and compared them using the 3D stem cell colony forming assay [29]. Parallel culture wells were treated with vehicle, H_2_O_2_ (as a source of ROS and oxidative stress), and/or the Aldh2 agonist, Alda-1. ROS treatment resulted in CFE reduction, while Alda-1 increased the CFE compared to that in the vehicle- and the ROS-treated wells (Fig. 6a-f). However, no significant difference in CFE was detected among the three groups of mice at the baseline or in response to ROS and/or Alda-1 treatments (Fig. 6g). Because fibroblasts from individuals with the ALDH2*2 polymorphism are known to be more vulnerable to injury than WT fibroblasts [39] and because fibroblasts are an essential component of the lung stem cell niche [29], we also investigated whether fibroblasts isolated from the lungs of WT, Aldh2*2 Tg, and *Aldh2* ^*−/−*^ mice have a different influence when co-cultured with WT tracheal basal stem cells in the colony forming assay. Co-culture with fibroblasts increased the CFE of WT tracheal basal stem cells, but no significant difference was observed among the three fibroblasts groups (Fig. 6h-l). Analysis of the differentiation profile in the grown colonies also revealed no significant difference.

### Aldh2*2 Tg mice show resistance to emphysema development in response to CS exposure compared to WT mice

Because the disturbed Aldh2 function in our mouse model revealed some functional effects on mitochondria, but not on stem cells in naïve mice, we wanted to compare them when they were exposed to some clinically-relevant injuries. We exposed the mice to chronic injury by CS, to acute injuries by H1N1 influenza viral infection, and to chemical injury using polidocanol.

**Fig. 7:**
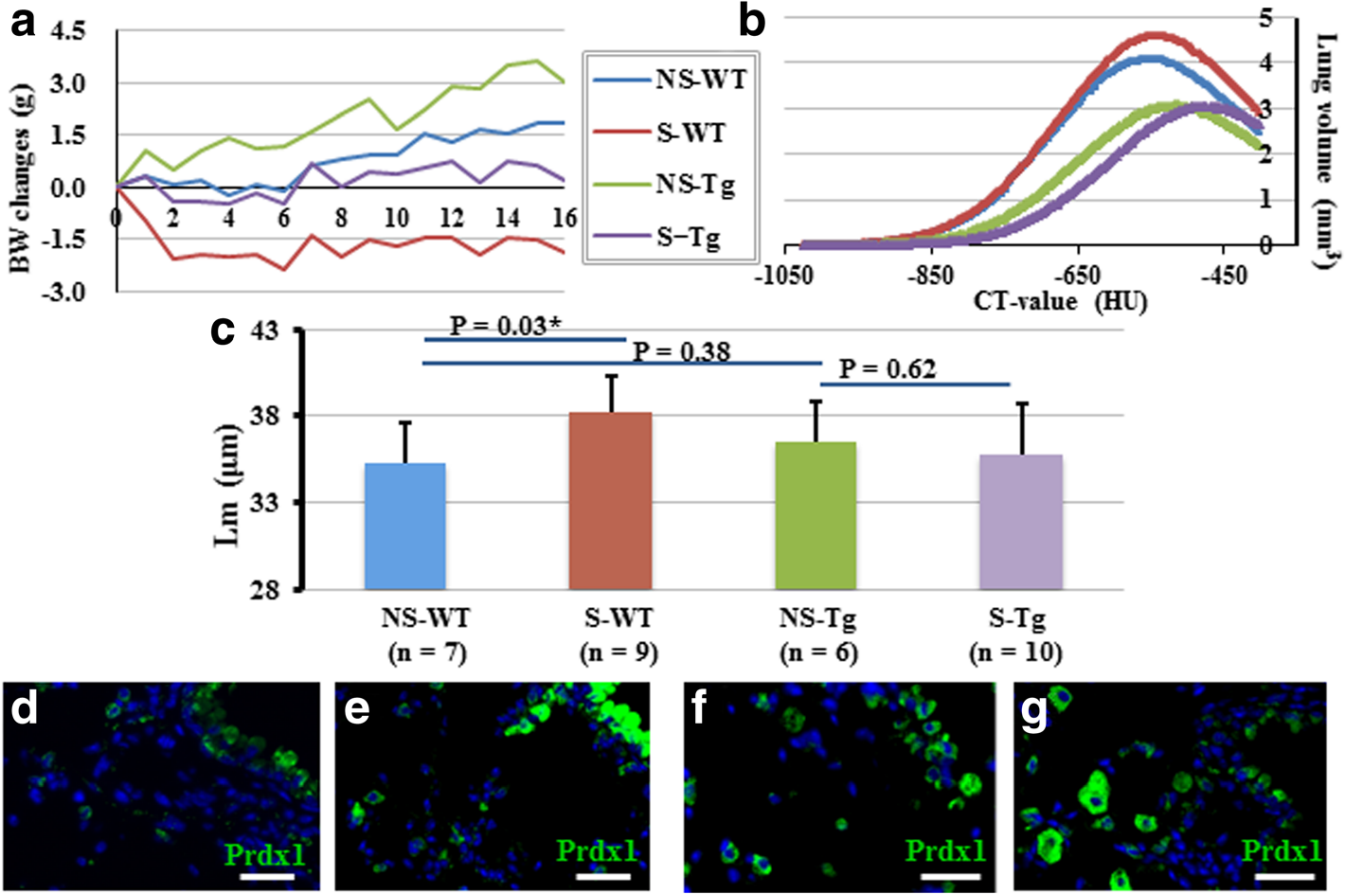
Effect of chronic injury with cigarette smoke on Aldh2*2 Tg and WT mice. **a** Effect of 16 weeks of CS (or sham) exposure on body weight (BW) of Aldh2*2 Tg mice and their WT littermates. **b** A representative graph showing the CT-calculated changes in lung volume versus CT-values in the non-smoking and smoking groups. **c** A graph showing the mean linear intercept (Lm) quantification of all histological analyses from all mice. NS: Non-smoking, S: smoking. **d**-**g** Representative immune fluorescence stained images showing that compared to naïve WT (**d**), naïve Tg mice express higher Prdx1 (**e**). CS exposure upregulated Prdx1 expression in both WT (**f**) and Tg (**g**). **d**-**g** Scale bars: 50 μm. Prdx1-green, Nuclei were stained with DAPI (*blue*) (**d**-**g**)

Aldh2*2 Tg mice and their WT littermates were exposed to CS for 16 weeks. Failure to gain weight compared to sham smoked controls is an established sign of the efficiency of the smoking protocol [40]. Indeed, both mice groups did not gain weight over the 16 weeks whereas the non-smoking control mice gained weight (Fig. 7a). All lungs were examined by micro-CT scanning followed by euthanasia and the lungs were collected and embedded in paraffin, to examine for digital and histological signs of lung damage and emphysema. Digital readings of the low attenuation area (LAA)%, CT values, and lung volumes were compared between the WT and Aldh2*2 Tg mice as previously described [31].

Although no change was detected in LAA%, the lung volume increased in WT mice (Fig. 7b) and mean linear intercepts (Lm) showed a significant increase in WT mice (Fig. 7c). These data suggest that mild emphysema developed at 16 weeks of CS exposure in WT mice as observed in the previous report [31]. On the other hand, we found that CS exposure neither increased the lung volume nor Lm in Aldh2*2 Tg mice (Fig. 7b-c) suggesting that the Aldh2*2 Tg mice are resistant to emphysema development in response to CS exposure.

As several mechanisms have been identified to mediate the pathogenesis of CS-induced emphysema, we examined several potential culprits. Mice without neutrophil elastase or IL-1 receptor did not develop CS-induced emphysema [41, 42] while mice with impaired expression of antioxidant genes developed more severe emphysema [43]. We used immunostaining to examine both naïve and CS-exposed WT and Aldh2*2 Tg mice for neutrophil infiltration, IL-1β expression and expression of the anti-oxidant Prdx1. In both WT and Aldh2*2 Tg mice, CS upregulated IL-1β and increased the number of neutrophils in the lungs. However, there were no significant differences between WT and Aldh2*2 Tg mice before or after CS-exposure (data not shown). Interestingly, naïve Aldh2*2 Tg showed higher expression of Prdx1, especially in airways. However, CS similarly induced Prdx1 expression in both WT and Aldh2*2 Tg mice (Fig. 7d-g).

### *Aldh2*^*−/−*^ mice sustain more extensive damage by influenza virus infection compared to WT mice

**Fig. 8:**
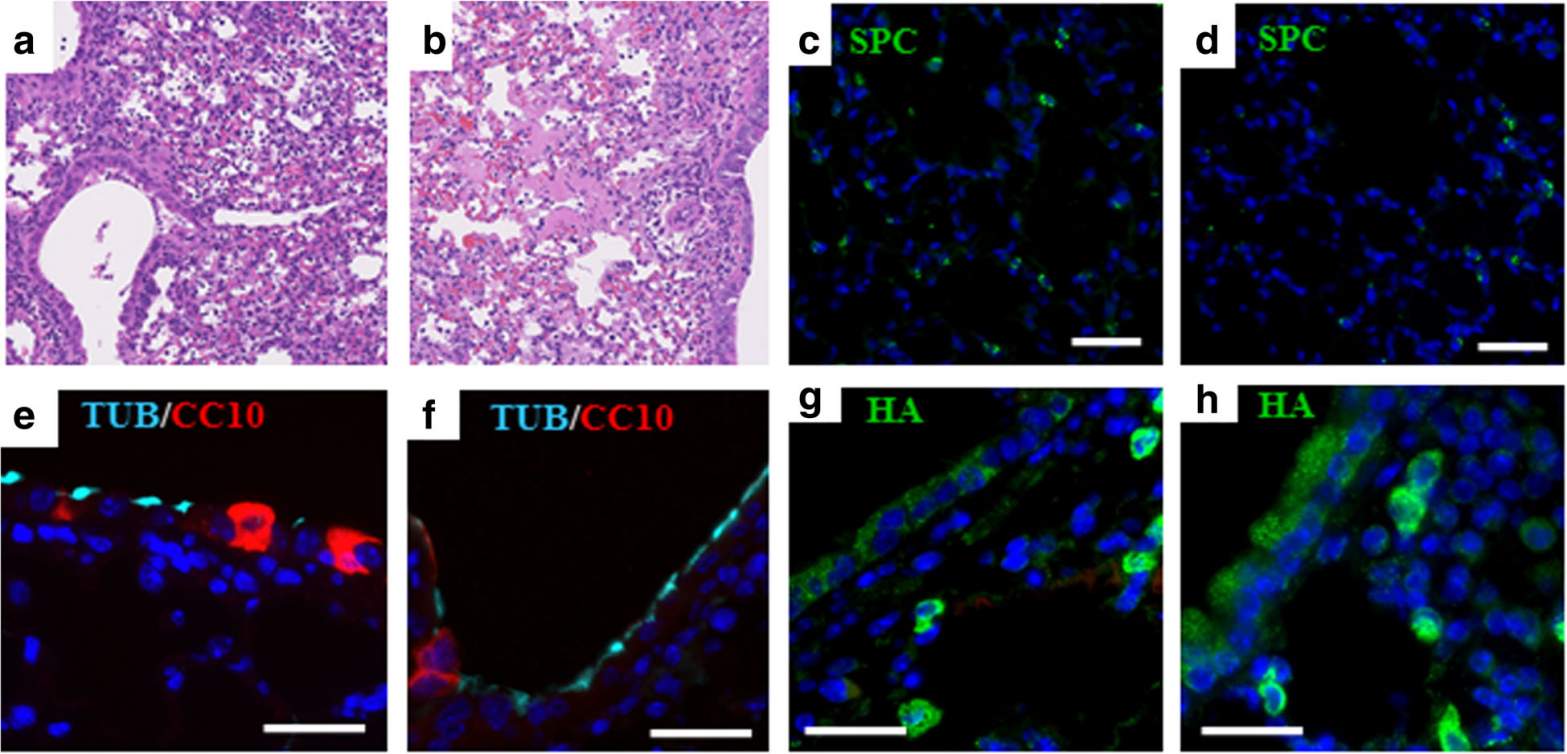
Effect of acute injury with H1N1 influenza infection on WT and *Aldh2* ^*−/−*^ mice. WT (*n =* 8) and *Aldh2* ^*−/−*^ (*n =* 7) mice were inoculated with H1N1 virus and observed for 8 days. Representative H&E images from H1N1-infected mouse lungs showing extensive inflammatory cell infiltration and pneumonia in both WT (**a**) and *Aldh2* ^*−/−*^ (**b**) lungs. Representative immune fluorescence stained images showing areas of damaged epithelial cells in the lung parenchyma in WT (**c**) and *Aldh2* ^*−/−*^ (**d**) lungs. (note the presence of few alveolar type II cells, (SPC-green)). The damage is also observed in the airway lining (note the reduced nuclear density, fewer club cells (CC10-red), and short ciliated cells (*β*-tubulin-cyan)) in WT (**e**) and *Aldh2* ^*−/−*^ (**f**). Viral load comparison between WT (**g**) and *Aldh2* ^*−/−*^ (**h**) mice by staining for hemagglutinin (HA-green), showed no difference. Scale bars: 50 μm. Nuclei were stained with DAPI (*blue*) (**c**-**h**)

WT and *Aldh2* ^*−/−*^ mice were infected with H1N1 influenza through the nose and lungs were collected after 8 days. There was no difference in survival or body weight loss. All lungs were examined histologically, and extensive inflammatory cell infiltration and pneumonia were obvious in all mice with no significant difference between the WT and *Aldh2* ^*−/−*^ (Fig. 8a-b). However, when we quantified the epithelial cell types by immunostaining, an average of only 70.6% of the lung area in WT mice, but 80.6% in *Aldh2* ^*−/−*^ mice, showed complete loss of AT-II cells (Fig. 8c-d). Similarly, loss of club cells in the airways was more extensive in *Aldh2* ^*−/−*^ mice (58%) than in WT mice (51.9%) (Fig. 8e-f). Viral load was confirmed using an immunostaining for hemagglutinin, a class I viral fusion protein from the influenza virus’ envelope proteins and we found no significant difference between WT and *Aldh2* ^*−/−*^ mice (Fig. 8g-h).

### WT, Aldh2*2 Tg, and *Aldh2*^*−/−*^ mice show similar efficient airway repair after polidocanol injury

**Fig. 9:**
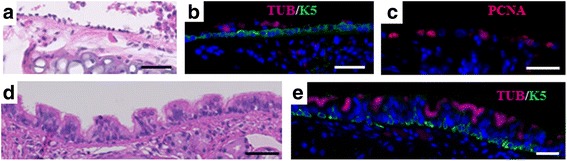
Effects of acute injury with polidocanol to tracheal epithelium of Aldh2*2 Tg, *Aldh2* ^*−/−*^ *,* and WT mice. **a**-**b** At 12 h after injury, most tracheal epithelial cells slough off and only a few K5+ basal cells survived. **c** At 48 h, these surviving basal cells are positive for PCNA. **d**-**e** At day 7, near complete repair is similarly observed in WT, Aldh2*2 Tg, and *Aldh2* ^*−/−*^ mice. The images shown are representative images from an *Aldh2* ^*−/−*^ mouse. *n =* 3–4/time point/genotype, Scale bars: 50 μm. Nuclei were stained with DAPI (*blue*) (**b**, **c**, **e**)

We also optimized the polidocanol injury protocol as described in the methods section. We found that administration of 10 μL of 2% polidocanol using tracheal intubation through a neck incision under anesthesia by isoflurane inhalation with continuous O_2_ resulted in the best survival and consistency. We administered polidocanol to the WT, Aldh2*2 Tg, and *Aldh2* ^*−/−*^ mice, and then collected samples at 12 h, 24 h, 48 h, day 5, and day 7 (*n =* 3–4/time point/genotype) for histological analysis. At 12 and 24 h, most epithelial cells had sloughed, leaving only few basal cells attached to the basement membrane (Fig. 9a-b). At 48 h, the surviving basal cells were actively proliferating as indicated by their positive staining for PCNA (proliferating cell nuclear antigen) (Fig. 9c). At day 5 and more-clearly at day 7, the epithelium was returning to the pseudo-stratified arrangement with ciliated and club cells differentiating from the proliferating basal cells (Fig. 9d-e). There was no significant difference among the groups at any time points. Although the Aldh2*2 Tg and *Aldh2* ^*−/−*^ mice had fewer tracheal epithelial basal cells than the WT mice in the steady state, the function of their basal cells in repairing the epithelia after polidocanol injury was as efficient as that in WT mice on day 7.

## Discussion

In this study, we have examined the potential effects of the common loss-of-function polymorphism in the human *ALDH2* gene on the lungs. We found that human carriers of the loss-of-function ALDH2*2 allele presented significantly lower FEV1/FVC than individuals who have the *ALDH2/ALDH2* allele, but that was not accompanied with lower predicted FEV1%, faster rate of annual FEV1 decline, or asthma/COPD. The mechanism resulting in this isolated functional impairment remains unclear. However, our results suggest that, in addition to the ALDH2 loss of function, the presence of several other genetic and environmental influences is required to inflect a clinically observable obstructive lung disease.

We expected that the lung cells might compensate for lost ALDH2 function by upregulating other ALDHs and/or antioxidants. However, surprisingly, *NRF2*, the key transcription factor regulating antioxidant gene expression, as well as one of the four antioxidant genes examined were significantly lower in the ALDH2*2 human samples than in the *ALDH2* group, and no significant change was detected in the expression of other ALDHs. Previous studies suggested that the non-functional ALDH2*2 may inactivate not only ALDH2, but also other structurally-similar ALDHs by forming hetero-tetramers [27]. Our results point to a previously unknown effect of the ALDH2 polymorphism, a negative effect on the antioxidant system.

In the mice experiments, we used two different types of mice that have some basic differences, which might explain some of the differences observed in their lung phenotypes. Aldh2*2 Tg mice have smaller body size compared to *Aldh2* ^*−/−*^ mice due to reduced muscle and fat masses. This suggests that the phenotypes observable in the Aldh2*2 Tg mice are not caused by the simple lack of Aldh2 activity. It was suggested that the presence of the non-functional Aldh2*2 enzyme in the cell interferes with the ability of other Aldhs to detoxify aldehydes. Indeed, when the aldehyde reductase activity was compared between these two mice, *Aldh2* ^*−/−*^ mouse displayed impaired Aldh activity only against acetaldehyde (as expected) and hexanal, while the Aldh2*2 Tg mouse showed impaired Aldh activity against most aldehydes examined [27]. We detected a histological abnormality in the tracheal epithelium in both Aldh2*2 Tg and *Aldh2* ^*−/−*^ mice. Interestingly, these abnormalities (thinning of the epithelium and decrease in cellular density most probably due to decrease in the number of basal cells) were not detectable at birth and only began to appear when the mice reached adulthood. Similar abnormalities (decrease in cellular density and basal cells) were recently described in old WT mice as “age-related changes” [44]. Indeed, our aged WT mice also showed these age-related changes. Accordingly, the differences in their tracheal cellular density and basal cell ratio in comparison with those of old Aldh2*2 Tg and *Aldh2* ^*−/−*^ mice became insignificant. This suggests that the *Aldh2* loss-of-function polymorphism induced a kind of accelerated aging in the airway epithelium. Similar histological abnormality could not be identified in the human samples, probably because of the small number of samples examined and that all samples were from aged individuals who have been exposed to multiple environmental confounding factors.

Another significant finding of this study is the detection of several abnormalities in the mitochondria of Aldh2*2 Tg and *Aldh2* ^*−/−*^ mice. We detected a marked increase in morphologically abnormal mitochondria in the club and basal cells of *Aldh2* ^*−/−*^ mice and a moderate increase in the Aldh2*2 Tg adult mice. The number of these abnormal mitochondria was also moderately higher in aged WT mice than in the adult WT mice, again suggesting the premature appearance of aging-related changes as a result of Aldh2 loss. Mitochondrial function has long been recognized to decline during aging, concomitant with the appearance of alterations in mitochondrial morphology, e.g., abnormally rounded mitochondria in aged mammals [45]. Therefore, on finding the morphologically abnormal mitochondria, we expected to also detect impairment of mitochondrial function. Thus, it was not surprising that the *Aldh2* ^*−/−*^ tracheal and lung cells presented fewer (functional) mitochondria and higher mitochondrial ROS. However, the finding that Aldh2*2 Tg mice have more mitochondria than the WT mice and showed no increase in mitochondrial ROS was both surprising and intriguing. The increase in the number of mitochondria and the upregulation of the antioxidant Prdx1 in Aldh2*2 Tg mice might also explain its resistance to CS-induced emphysema compared to the WT mice. It is possible that the lung cells in these mice developed some sort of “tolerance” pathway to acclimatize to the absence of Aldh2 function, similar to the pathway described in the heart for cardioprotection against aldehydes [27]. Further intensive investigations are needed to explain why the presence of non-functional Aldh2*2 in Aldh2*2 Tg mice (but not the absence of Aldh2, as in *Aldh2* ^*−/−*^ mice) results in such a differential effect on mitochondria. This points to the fact that results obtained from genetically engineered animals should not be automatically extrapolated to humans because pathological conditions in humans develop under the influence of an enormous unknown genetic and environmental variables.

To investigate a potential effect on stem cell function, we examined both airway and distal lung epithelium of both our mouse and human samples in our standardized in vitro CFE. No significant differences were detected among groups in mice or human, neither in the comparison of epithelial stem cells nor in their supportive (niche) fibroblast cells. This is similar to what has been described for the aging effect on mouse tracheal basal stem cells: despite the decrease in their number with age, in vitro CFE is preserved [41].

Each injury model examined in this study showed a different trend. Chronic exposure to CS resulted in development of signs suggestive of emphysema in WT, but not in Aldh2*2 Tg mice. The ALDH2*2 allele is a risk factor for the development of many diseases and cancers [16–19], especially when combined with alcohol consumption. On the other hand, it provides a protective effect against alcoholism and alcohol-induced diseases, obviously by inducing a behavioral change among individuals carrying the polymorphism causing them to abstain from alcohol consumption to avoid the unpleasant symptoms of flushing and severe hangovers [46, 47]. However, a recent report suggested that the ALDH2*2 allele provided an alcohol consumption-independent protection against vascular stenosis [48]. In this study, CS exposure increased the lung volume in WT mice but not in Aldh2*2 Tg mice. On the other hand, the CT value was increased in Aldh2*2 Tg mice at 16 weeks. The same phenomenon was temporarily observed in WT mice until 12 weeks of CS, before development of emphysema in our previous study [31]. These data imply that the Aldh2*2 loss-of-function polymorphism protects against development of emphysema in response to chronic CS. More experiments are needed to elucidate the exact role of ALDH2 in the development of emphysema.

Further, there was no difference in the survival, body weight loss, inflammatory cell infiltration, and pneumonia between the WT and *Aldh2* ^*−/−*^ mice infected with H1N1 influenza virus. However, the *Aldh2* ^*−/−*^ mice displayed a more extensive loss of airway and lung parenchymal epithelium than the WT mice did. A genetic association study on a large number of individuals with influenza infection is needed to compare the severity of symptoms, the duration to recovery, and extent of lung damage between the patients with *ALDH2* versus ALDH2*2 genotypes to confirm these animal findings.

Finally, no difference in repair efficiency was observed between the WT and the Aldh2*2 Tg or the *Aldh2* ^*−/−*^ mice following polidocanol injury. Collectively, the interaction and the role played by ALDH2 in the lung cells seems complex and the outcome depends on the cumulative interaction between the environment and the type of injuries/disease to which the lungs are exposed throughout the lifetime of the individual. Future studies are warranted to elucidate the interaction of ALDH2 enzyme and the environmental insults specific to the lung, like air pollution and CS, as well as ALDH2-specific insults associated with alcohol consumption.

## Conclusion

This is the first study to extensively characterize the effects of the common human loss-of-function polymorphism in the *ALDH2* gene on the lungs. We analyzed data from large human cohorts of healthy individuals, patients with bronchial asthma and COPD, as well as lung surgical samples. We also used two different models of genetically manipulated mice to mimic the effect of the *ALDH2* polymorphism in humans. We conclude that the *ALDH2* loss-of-function polymorphism exerts several subtle effects on the lungs. Some of which are similar to changes observed during normal aging of the lungs.

## Abbreviations

ALDH: Aldehyde dehydrogenases
COPD: Chronic obstructive pulmonary disease
CS: Cigarette smoke
FEV1: Forced expiratory volume in 1 second
FVC: Forced vital capacity
LAA: Low attenuation area
Lm: Mean linear intercepts
MTEC: Mouse tracheal epithelial cells
RBC: Red blood cell
ROS: Reactive oxygen species
Tg: Transgenic
WT: Wild type
